# OrchidBase 6.0: increasing the number of *Cymbidium* (Orchidaceae) genomes and new bioinformatic tools for orchid genome analysis

**DOI:** 10.1186/s12870-024-06024-1

**Published:** 2025-01-02

**Authors:** You-Yi Chen, Ye Sun, Chung-I Li, Shao-Ting Lin, Hao-Chen Zheng, Zhe-Bin Zhang, Bing-Ru Lee, Chun-Lin Hsieh, Yu-Yun Hsiao, Chi-Nga Chow, Chien-Wen Yang, Wen-Chi Chang, Heming Chen, Feng-Xi Yang, Gen-Fa Zhu, Qinyao Zheng, Cheng-Yuan Zhou, Zhuang Zhao, Ye Ai, Lin-Ying Wang, Deqiang Chen, Xin He, Ming-Zhong Huang, Dong-Hui Peng, Hao Yu, Siren Lan, Zhong-Jian Liu, Wei-Sheng Wu, Wen-Chieh Tsai

**Affiliations:** 1 Department of Agronomy, National Chiayi University, Chaiyi, 600 Taiwan; 2 Jiangsu Lixiahe District Institute of Agricultural Sciences, Yangzhou, 225007 China; 3 Department of Statistics, National Cheng Kung University, Tainan City, 701 Taiwan; 4 Graduate Program in Translational Agricultural Sciences, National Cheng Kung University and Academia Sinica, Tainan City, Taiwan; 5 Department of Electrical Engineering, National Cheng Kung University, Tainan City, 701 Taiwan; 6 Orchid Research and Development Center, National Cheng Kung University, Tainan City, 701 Taiwan; 7 Institute of Tropical Plant Sciences and Microbiology, National Cheng Kung University, Tainan City, 701 Taiwan; 8 Guangdong Key Laboratory of Ornamental Plant Germplasm Innovation and Utilization, Institute of Environmental Horticulture, Guangdong Academy of Agricultural, Guangzhou, 510640 China; 9 Key Lab of National Forestry and Grassland Administration for Orchid Conservation and Utilization and International Orchid Research Center, College of Landscape Architecture, Fujian Agriculture and Forestry University, Fuzhou, Fujian 350002 China; 10 College of Forestry, Fujian Agriculture and Forestry University, Fuzhou, 350002 China; 11 Department of Biological Sciences, National University of Singapore, Singapore, 117543 Singapore; 12 Department of Life Sciences, National Cheng Kung University, Tainan City, 701 Taiwan; 13 University Center for Bioscience and Biotechnology, National Cheng Kung University, Tainan City, 701 Taiwan

**Keywords:** Orchid, OrchidBase, Cymbidium, Whole-genome sequencing, Genome, Transcriptome

## Abstract

**Background:**

Orchids are well-known for their rich diversity of species as well as wide range habitats. Their floral structures are so unique in angiosperms that many of orchids are economically and culturally important in human society. Orchids pollination strategy and evolutionary trajectory are also fantastic human for centuries. Previously, OrchidBase was created not only for storage and management of orchid genomic and transcriptomic information including *Apostasia shenzhenica*, *Dendrobium catenatum*, *Phalaenopsis equestris*, and two species of *Platanthera* that belong to three different subfamilies of Orchidaceae, but explored orchid genetic sequences for their function. The OrchidBase offers an opportunity for the plant science community to compare orchid genomes and transcriptomes, and retrieve orchid sequences for further study.

**Description:**

Recently, three whole-genome sequences of the Epidendroideae species, *Cymbidium sinense*, *C. ensifolium* and *C. goeringii*, were sequenced *de novo*, assembled, and analyzed. In addition, the systemic transcriptomes of these three species have been established. We included these datasets to develop a new version of OrchidBase 6.0. Furthermore, four new analytical methods, namely regulation, updated transcriptome, advanced BLAST, and domain search, were developed for orchid genome analyses.

**Conclusion:**

OrchidBase 6.0 extended genetic information to that of eight orchid species and created new tools for an expanded community curation in response to the ever-increasing volume and complexity of data.

**Supplementary Information:**

The online version contains supplementary material available at 10.1186/s12870-024-06024-1.

## Introduction

*Cymbidium* contains approximately 80 species and belongs to the subfamily Epidendroideae of the family Orchidaceae. This genus is distributed in tropical and subtropical Asia (northern India, China, Japan, Malaysia, the Philippines, and Borneo) and further south in Papua New Guinea and Northern Australia [1, 2]. The cultivation of *Cymbidium* can be traced back to the time of Confucius, approximately 2,500 years ago (B.C. 500). Through the accumulation of cultural and scientific development over several thousand years, *Cymbidium* species and hybrids have become one of the most commercially important orchids, not only in the floriculture industry but also in medicinal applications globally. *Cymbidium* shows diversified lifestyles for adaptation to the environment, including epiphytic; lithophytic; terrestrial; and rarely, leafless mycoheterotrophy lifestyles [3, 4]. *C. goeringii*, *C. ensifolium*, and *C. sinense* are terrestrial plants that are the most popular flowering ornamental orchids and are widely cultivated for their beauty and fragrance [5]. Therefore, the genome sequences of these three *Cymbidium* species have been selected for decoding and used to explore the molecular mechanisms of flowering, floral shape morphogenesis, and flower odor biosynthesis [6–8].

OrchidBase was created for the storage, management, and efficient usage of orchid genetic information. The data were primarily generated using first-generation sequencing technology. Sanger sequencing was performed on samples derived from *Phalaenopsis* reproductive organs [9]. OrchidBase 2.0 was constructed using transcriptomes derived from the floral buds of two species in each of the five subfamilies of Orchidaceae using next-generation sequencing (Solexa Illumina, San Diego, CA, USA) [10]. With the advancement in sequencing technology, the cost of sequencing has reduced, and the sequence production speed has greatly increased. As a result, and orchid whole-genome sequencing has been accomplished [6–8, 11–14]. Based on these orchid genomes and their transcriptomic sequences, OrchidBase has been updated with new sequences and newly developed tools for mining information embedded in these sequences [15–17]. In addition to OrchidBase, which provides orchid genomes and transcriptomes for analysis, several databases offer similar datasets and tools for mining specific orchid species, such as Orchidstra for *P. aphrodite* [18–20], OncidiumOrchidGenomeBase for *Oncidium* [21], and GelFAP for *Gastrodia elata* [22–25].

**Fig. 1:**
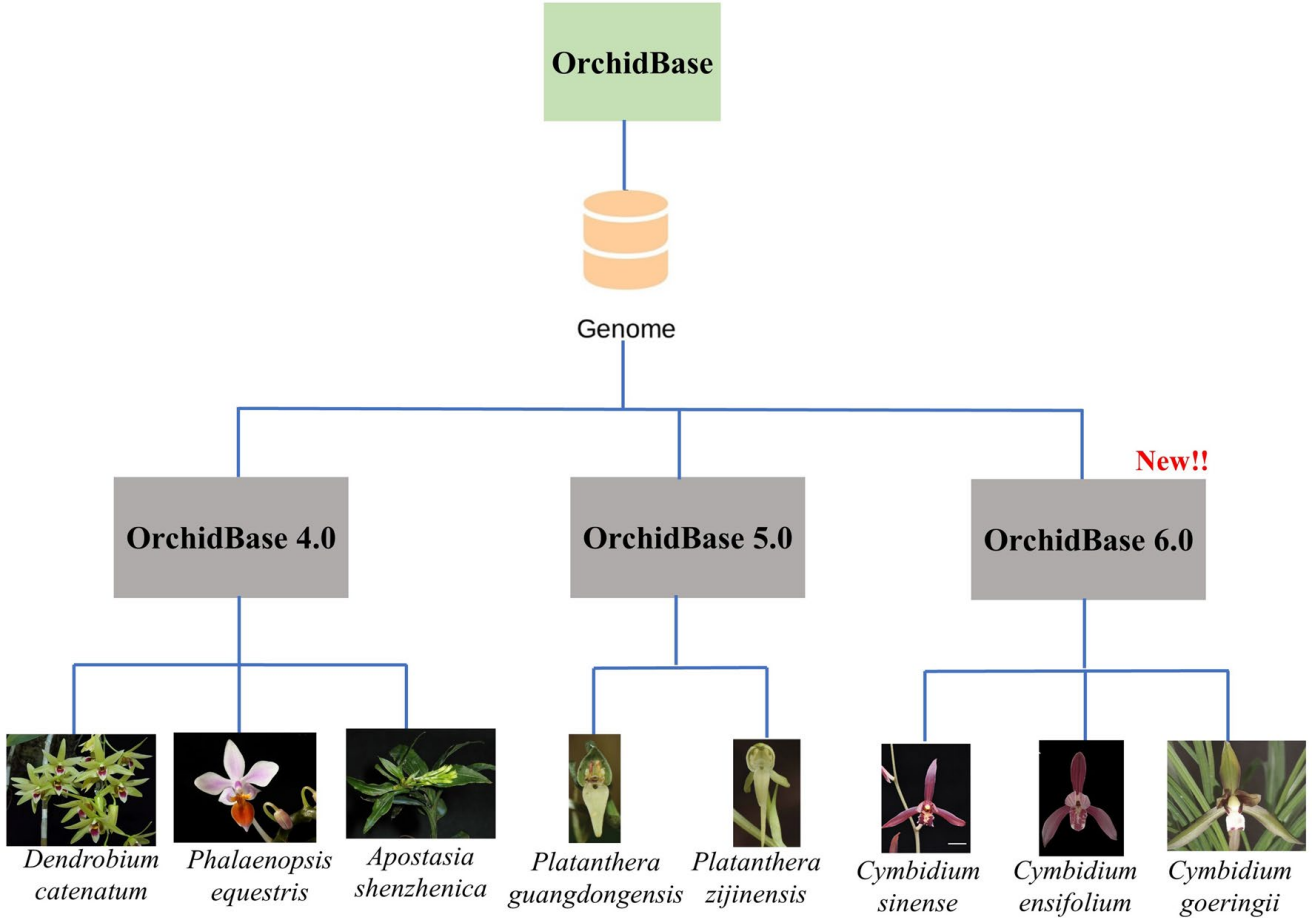
Genomic data from three *Cymbidium* species were added to OrchidBase 6.0. OrchidBase 6.0 includes genome information for eight orchid species. The pictures of the eight orchid species were from the authors

In OrchidBase 6.0, the genomes of three *Cymbidium* species, namely, *C. sinense*, *C. ensifolium*, and *C. gorengii*, belonging to the Epidendroideae family, and their relative transcriptomes derived from various floral developmental stages and tissues have been included (Fig. 1). Furthermore, the new tools, including transcription regulation analysis (promoters, transcription factors, and downstream targets), advanced transcriptome analysis, and advanced BLAST tools, have been developed for the functional analysis of orchid genes. The content of the OrchidBase 6.0 is summarized in Table 1. The information and tools launched in OrchidBase 6.0 are extensive and will be an excellent resource for orchid biology research.

**Table 1:** Summary of data and tools that could be browsed and used for the eight orchid species (Pha. Equestris, D. catenatum, Apo. Shenzhenica, P. zijinensis, P. guangdongensis, C. sinense, C. Ensifolium and C. Goeringii)

Transcriptome	Gene ID, FPKM and TPM values in various tissues
Genome browser	Scaffold ID, Scaffold sequence, Gene model, miRNA
Gene annotation	Gene ID, Gene sequence, BLAST top hit descriptions, KEGG pathway, GO terms, Interpro description, Swissprot description, TrEMBL description, miRNA
Metabolism pathway	Gene ID, Genes mapped to the KEGG pathways
Synteny	Gene ID, Physical positions of genes
Gene order	Gene ID, Physical positions of genes
miRNA-targets information	miRNA gene ID, Structure of miRNA, Target gene IDs of miRNA, Binding sites in the target genes of a miRNA
Regulation	Gene ID, Promoter binding site prediction
Tools	BLASTN, BLASTX, tBLASTX, BLASTP, tBLASTN, pfam ID, pfam description,

## Expanded database content

**Fig. 2:**
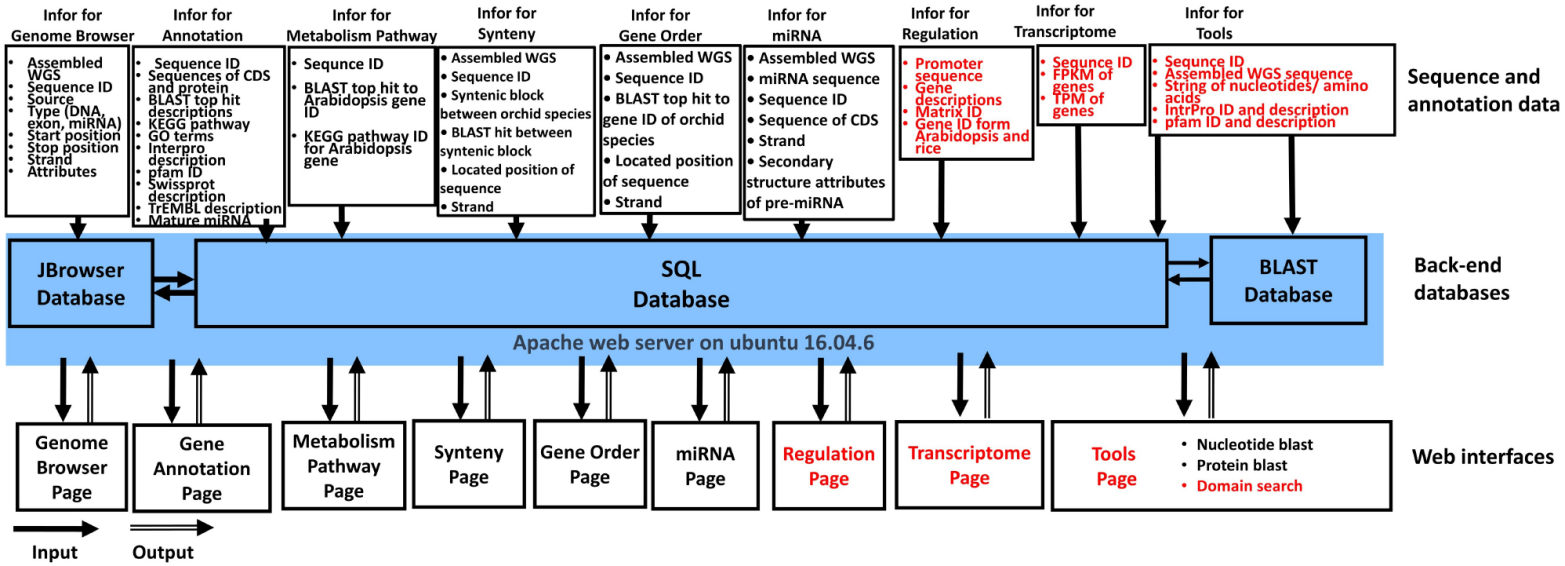
Overview of the OrchidBase 6.0 architecture

*C. sinense*, *C. ensifolium*, and *C. gorengii* each have a karyotype of 2 *N* = 2X = 40. We generated 429 Gb of data using Nanopore technology [26] and 670 Gb using Hi-C sequencing technology for *C. sinense*; 351 Gb using PacBio technology and 349 Gb using Hi-C technology for *C. ensifolium*; and 478 Gb using PacBio technology and 296 Gb using Hi-C technology for *C. gorengii*. The genome assemblies were 3.45 Gb, with a contig N50 value of 1.11 Mb; 3.63 Gb, with a contig N50 value of 1.21 Mb; and 4.07 Gb, with a contig N50 value of 1.04 Mb for the *C. sinense*, *C. ensifolium* and *C. gorengii* genomes, respectively (Table 2) [6–8]. Twenty pseudochromosomes were constructed for each *Cymbidium* species based on the assembled sequences. The raw data and whole-genome-assembled scaffold sequences of *C. sinense* and *C. gorengii* were downloaded from BioProject PRJNA743748 and PRJNA749652, respectively, and deposited in the National Center for Biotechnology Information database. The corresponding data for *C. ensifolium* (BioProject/GSA PRJCA005355/CRA004327) was downloaded from the National Genomics Data Center. The statistics for the added orchid genomes are presented in OrchidBase 6.0 (http://cosbi.ee.ncku.edu.tw/orchibase6/). Based on these datasets, 29,638, 29,073, and 29,272 protein-coding genes were predicted for the genomes of *C. sinense*, *C. ensifolium*, and *C. gorengii*, respectively. Furthermore, 200, 71, and 147 miRNA candidates have been identified in the *C. sinense*, *C. ensifolium*, and *C. gorengii* genomes, respectively [6–8]. Each predicted gene and miRNA were assigned a specific ID. Specific genes or miRNAs can be selected to investigate their annotated functions associated with biological processes. The information required for the new developed tools is illustrated by red color in the Figure 2.

**Table 2:** Comparisons of the assembled genomes among eight orchid species in the OrchidBase 6.0

Orchid species	Assembled genome size	N50 length of Scaffold (Mb)	N50 length of contig size	Number of predicted genes	Reference
*Phalaenoipsis equestris*	1.03 Gb	1.22	45.8 Kb	29,545	Zhang et al. 2017
*Dendrobium catenatum*	1.12 Gb	1.06	51.7 Kb	29,257	Zhang et al., 2017
*Apostasia shenzhenica*	349 Mb	3.03	80.1 Kb	21,841	Zhang et al., 2017
*Platanthera zijinensis*	4.19 Gb	nd	1.77 Mb	24,513	Li et al., 2022
*Platanthera guangdongensis*	4.20 Gb	nd	1.57 Mb	22,559	Li et al., 2022
*Cymbidium sinense*	3.45 Gb	nd	1.11 Mb	29,638	Yang et al., 2021
*Cymbidium ensifolium*	3.63 Gb	nd	1.21 Mb	29,073	Ai et al., 2021
*Cymbidium goeringii*	4.07 Gb	nd	1.04 Mb	29,272	Ye et al., 2021

nd: not determined

The transcriptomic data derived from the three *Cymbidium* species were downloaded from BioProjects PRJNA743748 (*C. sinense*), PRJNA749652 (*C. gorengii*) and BioProject/GSA PRJCA005355/CRA004327 (*C. ensifolium*). All RNA sequencing reads were mapped to the predicted genes and calculated as transcripts per million (TPM), fragments per kilobase of transcript per million mapped reads (FPKM), or raw counts for each gene in various tissues and at different developmental stages to provide the gene expression profiles. This biological information was integrated into the updated version of OrchidBase 6.0.

### Identification of transcription factor (TF) genes in the genomes of orchid species

To identify orchid genes encoding TFs, we retrieved the TF protein sequences of *Arabidopsis thaliana* and *Oryza sativa* subsp. *japonica* from PlantTFDB 5.0 (https://planttfdb.gao-lab.org/index.php). In total, 2,296 and 2,408 TF sequences from *A. thaliana* and *O. sativa* subsp. *japonica*, respectively, were used as queries in BLASTP searches against each of the predicted proteomes of eight orchids, with an E value of 10^− 5^ to obtain 13,169 putative orchid TF genes that could be categorized into different subfamilies (Supplementary Table 1). To predict TF binding sites, the region 2,000 bp upstream of the translation start site of each gene was annotated for each orchid species genome. These data were retrieved and searched using the Match™ program [27] based on the position weight matrices created in PlantPan 3.0 [28]. In total, 1,786 and 420 TFmatrixIDs were predicted for each orchid species using *Arabidopsis* and rice model plants, respectively (Table 3). Furthermore, approximately 37–77 million TF binding sites in the orchid genomes were predicted using the *Arabidopsis* matrix, and 15–28 million binding sites were predicted using the rice matrix (Table 3).

**Table 3:** The number of predicted TFmatrixID and TF binding site at the promoter of each orchid genome

Orchid species	Compared model plant species	Number of hit TFmatrix ID and TF binding site at the promoter of each orchid species	Number of TF binding sites at promoter
*Aps. shenzhenica*	*A. thaliana*	1,786	45,452,473
*P. zijinensis*	*A. thaliana*	1,786	51,027,954
*P. guangdongensis*	*A. thaliana*	1,786	37,226,223
*Pha. equestris*	*A. thaliana*	1,786	51,218,142
*D. catenatum*	*A. thaliana*	1,786	53,846,987
*C. sinense*	*A. thaliana*	1,786	65,068,952
*C. ensifolium*	*A. thaliana*	1,786	63,385,827
*C. goeringii*	*A. thaliana*	1,786	76,868,231
*A. shenzhenica*	*O. sativa*	420	16,847,177
*P. zijinensis*	*O. sativa*	420	21,110,631
*P. guangdongensis*	*O. sativa*	420	15,589,098
*Pha. equestris*	*O. sativa*	420	19,206,180
*D. catenatum*	*O. sativa*	420	19,977,158
*C. sinense*	*O. sativa*	420	23,942,903
*C. ensifolium*	*O. sativa*	420	23,455,231
*C. goeringii*	*O. sativa*	420	28,271,112

### Searching the genome information for the three species of *Cymbidium* in the database

**Fig. 3:**
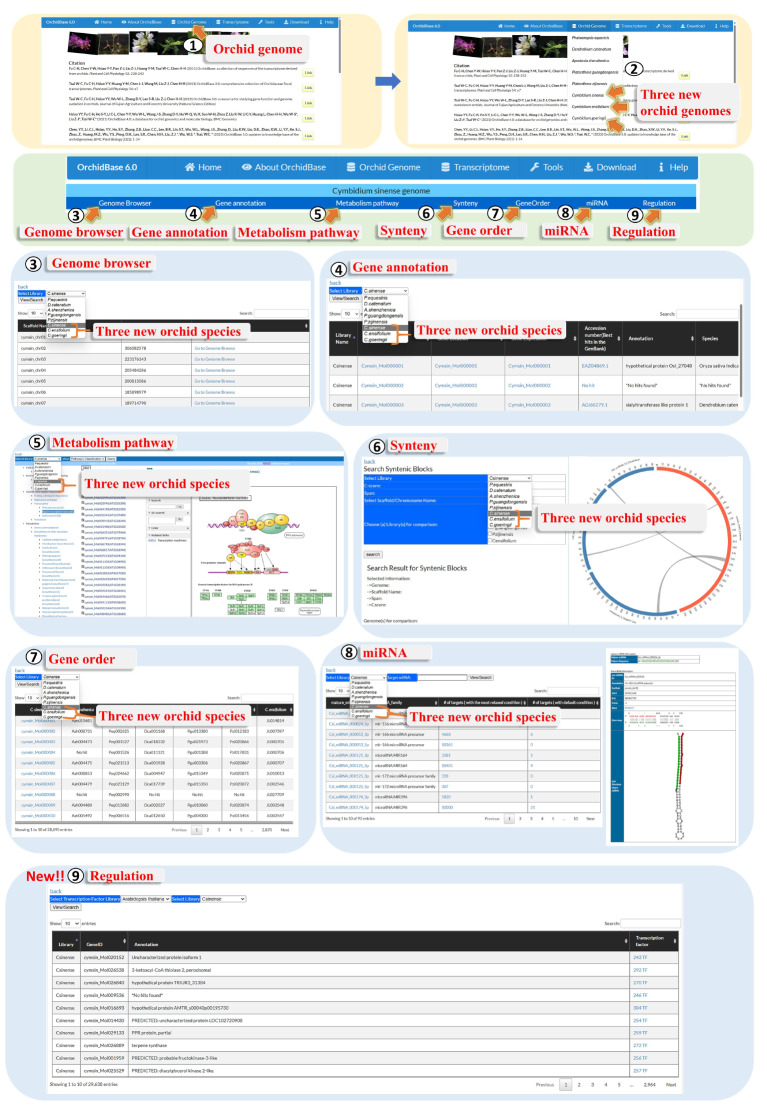
Genome page of OrchidBase 6.0. Three *Cymbidium* genomes were newly compiled in OrchidBase 6.0. Analytical tools such as a genome browser, gene annotation, metabolic pathways, synteny, gene order, miRNA, and regulation tools were developed

The genome information for the three *Cymbidium* species in OrchidBase 6.0 can be accessed using the assembled pseudochromosomes and predicted genes. Through the web interface, the newly added orchid genome information in OrchidBase 6.0 can be freely obtained. The information can be linked via the “Orchid Genome” icon (Fig. 3, Step 1). Using this interface, users are able to select one of the five existing orchid genomes (*Pha. equestris*, *D. catenatum*, *Aps. shenzhenica*, *P. zijinensis*, and *P. guangdongensis*), and from the three newly added *Cymbidium* genomes (*C. sinense*, *C. ensifolium*, *and C. gorengii*) (Fig. 3, Step 2). Users can then access the genome browser (Fig. 3, Step 3) and obtain information about gene annotation (Fig. 3, Step 4), metabolic pathways (Fig. 3, Step 5), synteny (Fig. 3, step 6), gene order (Fig. 3, Step 7), miRNAs (Fig. 3, Step 8), and regulation (Fig. 3, Step 9) by searching the orchid genome. Comparative analysis can then be performed using the selected orchid genomes. The genome browser and gene annotation, metabolic pathway, synteny, gene order, and miRNA information were introduced in the previous versions of OrchidBase [16, 17]. In the following sections, we explain in detail the new “Regulation” function, the updated transcriptome information, and advanced BLAST and Domain searches, which can be found in the Tools menu.

### Data content and “Regulation” web interface

**Fig. 4:**
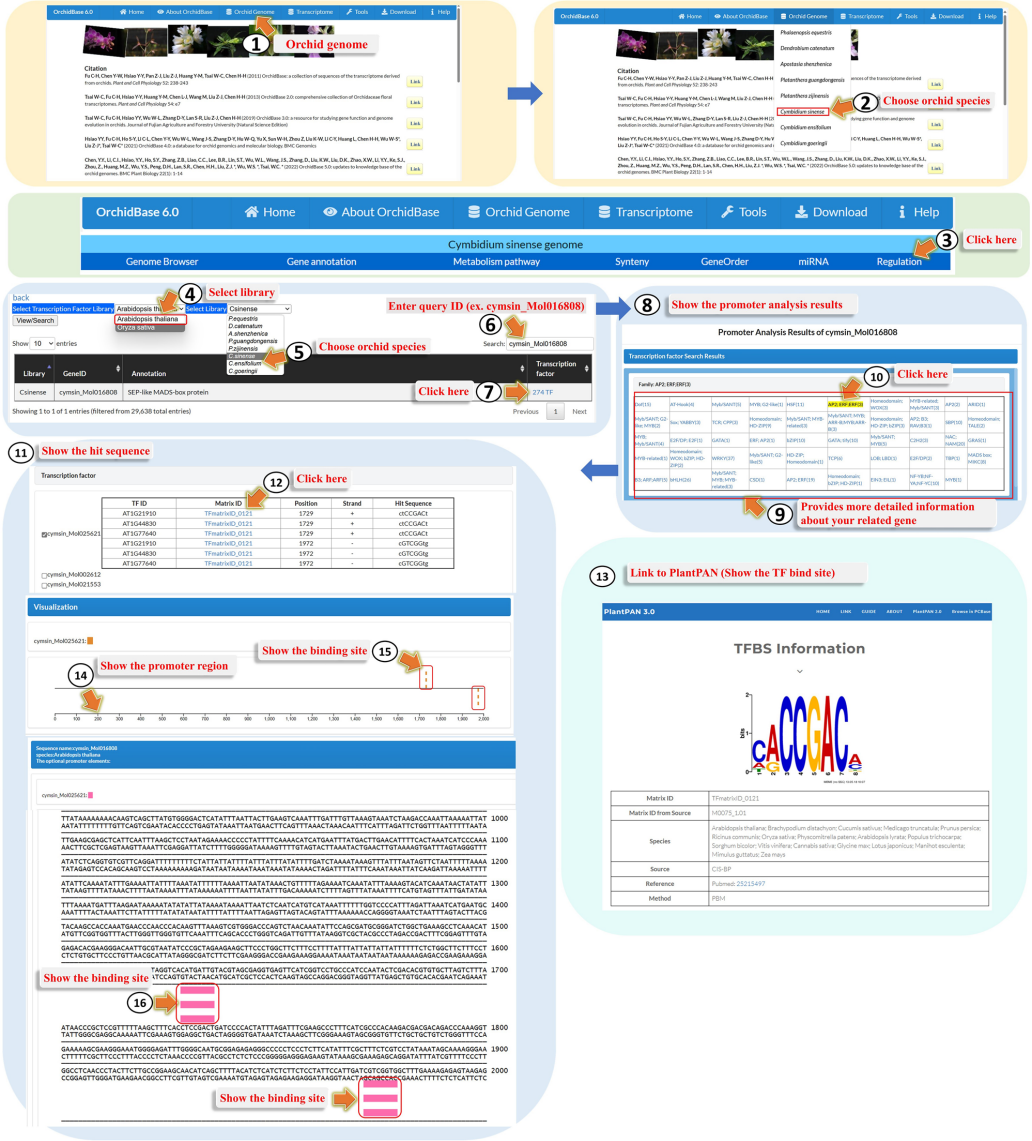
A step-by-step guide for using the “Regulation” tool

The OrchidBase 6.0 database update provides a “Regulation” function for each orchid genome. This function allows users to predict genes that may be regulated by different types of TFs and the binding sites at which the corresponding TFs bind to the promoters. Regulation analysis provides a graphical interface for displaying the relationships between genes, the binding sites and sequences at their individual promoters, and the corresponding TFs (Fig. 4). To use the Regulation Analysis page, users can click on the orchid genome (Fig. 4, Step 1) and choose one of the orchid species (Fig. 4, Step 2). They will then be navigated to the main function page for genome analysis and enter the “Regulation” page (Fig. 4, Step 3). On the “Regulation” page, users can select one of the TF reference libraries (*A. thaliana* or *O. sativa*) (Fig. 4, Step 4), which means that the identified TFs in each of the orchid genomes were based on orthologs in *Arabidopsis* or rice. One orchid species (Fig. 4, Step 5) can be chosen or maintained, as shown in Fig. 4, Step 2. If users are interested in gene ID analysis, they can fill in the “Search” box (Fig. 4, Step 6). In this study, the gene ID *cymsin_Mol016808*, a gene encoding *SEPTALLA* (*SEP*)-like MADS-box protein, was used as an example. The results for the example showed that 274 TFs could bind to the promoter of *cymsin_Mol016808*. By clicking on these 274 TFs (Fig. 4, Step 7), users can observe a table characterizing different TF families and the number of corresponding members that potentially regulate the expression of *cymsin_Mol016808* (Fig. 4, Steps 8 and 9). Users can choose any of the TF families, and AP2, ERF, and ERF (3) can be selected (Fig. 4, Step 10). On the same page, a new table under the TF families table shows the IDs of three genes encoding AP2, ERF, and ERF TFs that bind to specific sequences in the *cymsin_Mol016808* promoter, and the IDs of their ortholog genes in *Arabidopsis* (Fig. 4, Step 11). Clicking on the binding site directs users to PlantPAN 3.0, where they can see the TFmatrixID logo and access additional information related to the binding sites (Fig. 4, Steps 12 and 13). Under the TF table, users can further visualize a graph of the binding positions and sequences of the TF (Fig. 4, Steps 14–16). Different TFs are shown in different colors.

### Updated transcriptome

**Fig. 5:**
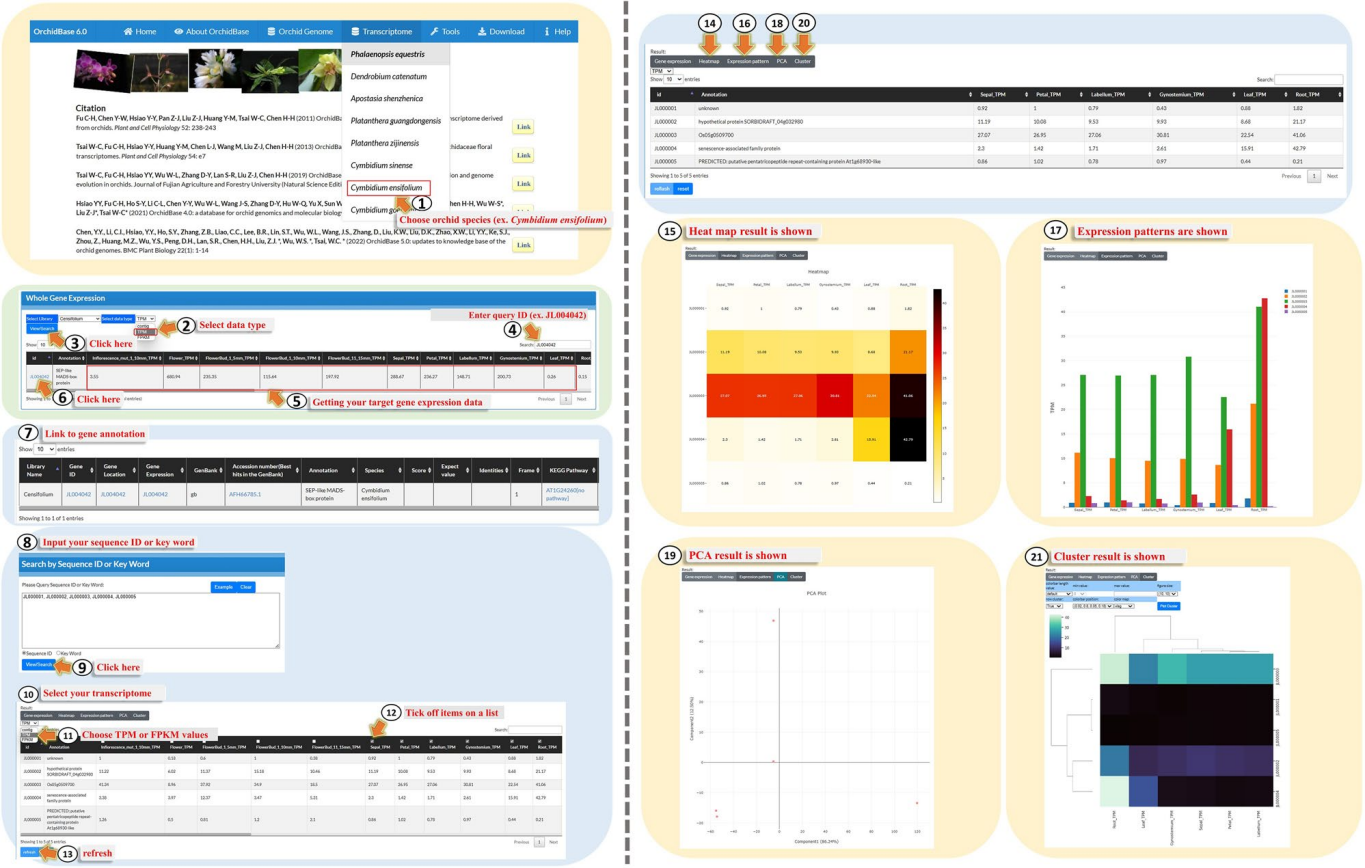
A step-by-step guide for using the updated “Transcriptome” tool

The previous version of the transcriptome in OrchidBase only provided the FPKM of each gene in each sequenced orchid genome [15–17]. In the current version, we provide the TPM and raw counts for the expression of each gene as well as different presentation styles for the gene expression data. The user can navigate to the transcriptome and choose one of the orchids (Fig. 5, Step 1). This page shows the expression of each gene in different tissues and organs. Users can select the data type with the contig (raw count), TPM, or FPKM (Fig. 5, Step 2), and then click “Search” (Fig. 5, Step 3). Alternatively, they can enter the gene ID, if they know it, in the “Search” box (Fig. 5, Step 4). The subsequent page then shows the expression patterns that the users would like to see (Fig. 5, Step 5). Users can further click “Gene ID” (Fig. 5, Step 6) to hyperlink to the gene annotation (Fig. 5, step 7). They can even type several Gene IDs or keywords in the “Search” box to explore multiple gene expression patterns (Fig. 5, Step 8). After clicking “View/Search” (Fig. 5, Step 9), users can obtain the transcriptome of assigned genes listed in the table under the “Search” box (Fig. 5, Step 10). Users can choose the values used to measure the expression levels (Fig. 5, Step 11) and further select items from various tissues or organs (Fig. 5, Step 12) to investigate their expression patterns. The button under the table is designed to “Refresh or Reset” (Fig. 5, Step 13). In addition to the table describing the expression patterns of the assigned genes, we designed several graphic modes to visualize the transcripts of the genes, including a heatmap (Fig. 5, Steps 14 and 15), bar chart (Fig. 5, Steps 16 and 17), principal component analysis (PCA) results (Fig. 5, Steps 18 and 19), and hierarchical clustering (Fig. 5, Steps 20 and 21). Overall, this page provides an interface for users to explore gene expression patterns using TPM, FPKM, or raw counts, and users can obtain gene annotations using the gene ID.

### Advanced BLAST

**Fig. 6:**
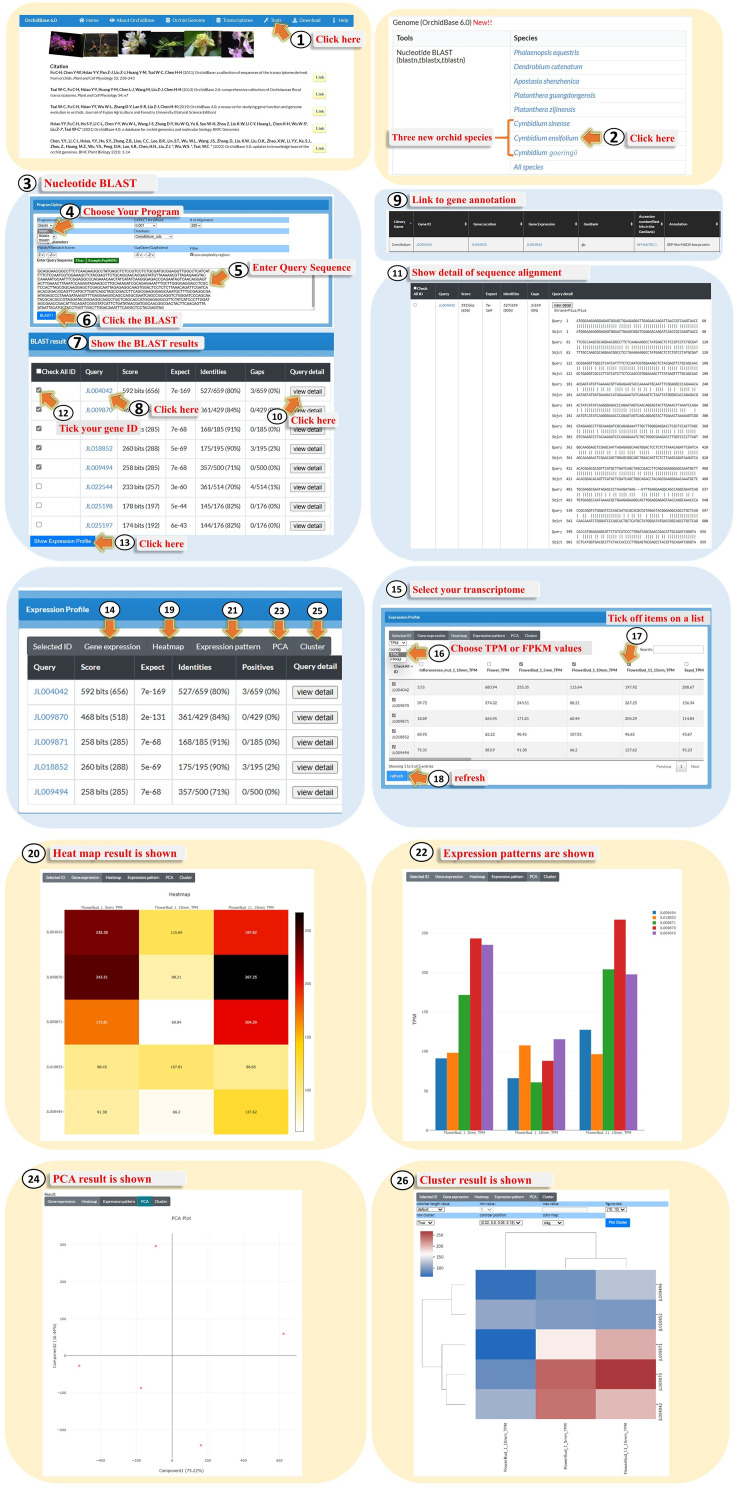
A step-by-step guide for using the advanced “BLAST” tool

BLAST is one of the most popular pairwise alignment tools to search for similar sequences stored in databases [29]. However, scientists would like to know the expression patterns of hit sequences to further infer their functions. Here, we combined the BLAST tool with different expression patterns to display tools that simultaneously reveal the biological significance of the genes. Users can visit “Tools” (Fig. 6, Step 1), and select one of the sequenced orchid genomes. Here, we selected *C. ensifolium* using nucleotide BLAST as an example (Fig. 6, Step 2). Based on the nucleotide BLAST search (Fig. 6, Step 3), users can select one of the nucleotide BLAST programs (Fig. 6, Step 4), paste the nucleotide sequence (Fig. 6, Step 5), and click BLAST (Fig. 6, step 6). In the BLAST results page (Fig. 6, Step 7), users would click the hit “Gene ID” (Fig. 6, Step 8) to link to the gene annotation (Fig. 6, Step 9), or click the “View Detail” icon to obtain the sequence alignment (Fig. 6, Steps 10 and 11). Additionally, users can tick any one of the hit gene IDs (Fig. 6, Step 12) and click “Show Expression Profile” at the bottom of the table (Fig. 6, Step 13). The subsequent page provides various graphic presentations of the updated transcriptomes described above (Fig. 6, Steps 14–26). In summary, this tool not only contributes to pairwise alignment results, but also provides additional gene annotation and expression profiles of the hit sequences.

### Domain search

**Fig. 7:**
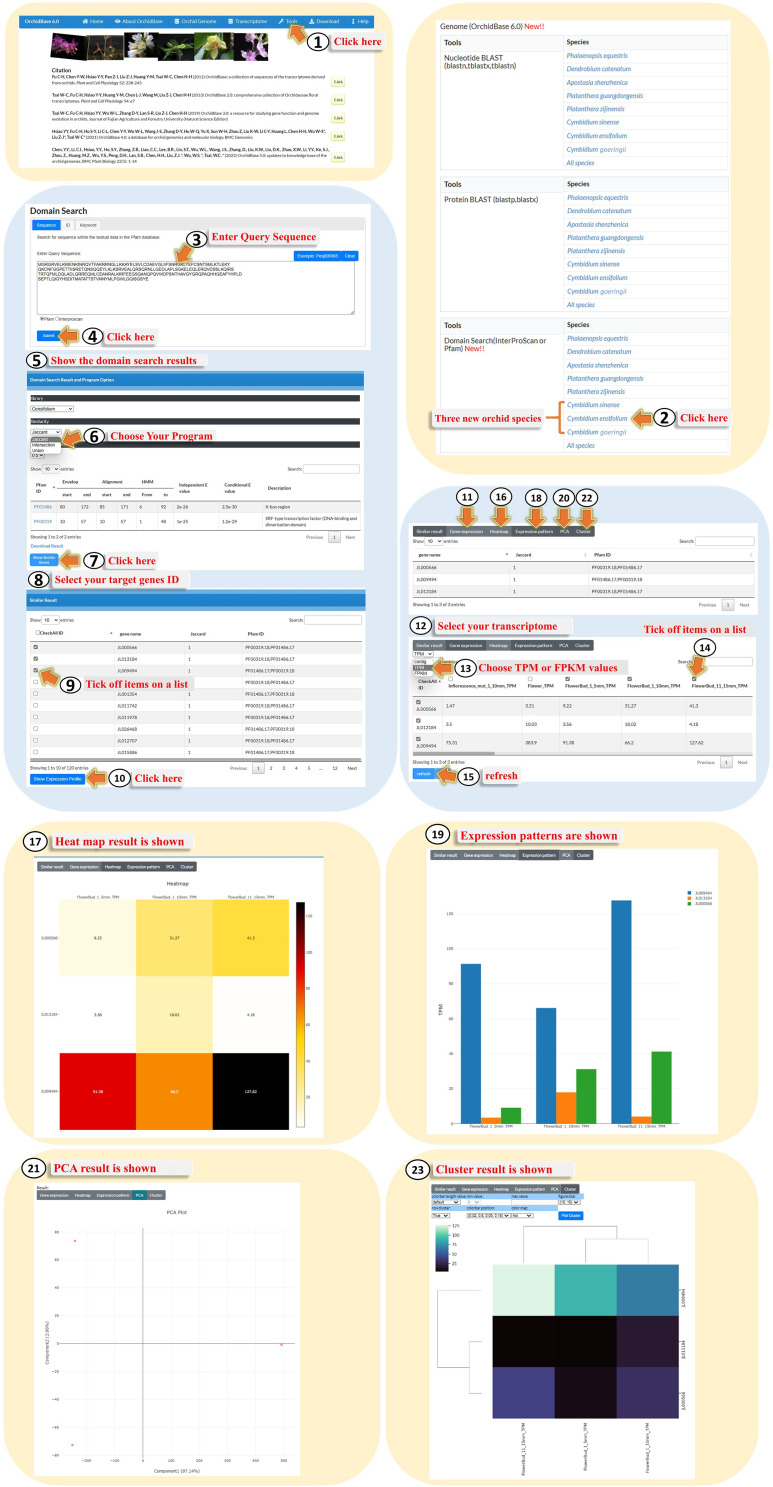
A step-by-step guide for using the advanced “Domain Search” tool

Protein domains are fundamental units of protein structure, folding, function, evolution, and design. They are considered homologous sequences encoded in different gene contexts that have remained intact at the sequence level throughout evolution. Based on these concepts, we designed the tool “Domain Search” to characterize the protein-coding sequences based on the Pfam and InterPro classifications. For example, with the “Domain Research” tool, users can click on “Tools” (Fig. 7, Step 1) and choose one of the orchid species in the panel of Tools_Domain Search (InterProScan or Pfam) (Fig. 7, Step 2). Protein sequences can be pasted in the box (Fig. 7, Step 3), and Pfam or Interproscan can be chosen. After clicking “Submit” (Fig. 7, Step 4), the page shows an additional table presenting the hit sequence ID in the genome of the selected species (Fig. 7, Step 5). The page also allows users to choose either the Jaccard, intersection, or union method (Fig. 7, Step 6) for similarity comparison and to determine how the domains are screened for the inclusion of the query. The unique design of this tool includes the “Domain Search” and the expression patterns of the hit sequences (Fig. 7, Step 7). Users can further click “Show Similar Gene” (Fig. 7, Step 8), tick the genes of interest (Fig. 7, Step 9), and click “Show Expression Profile” (Fig. 7, Step 10). The additional table page provides various graphical presentations of the expression pattern, such as the updated transcriptome described above (Fig. 7, Steps 11–23).

### A case of study – by using regulation

**Fig. 8:**
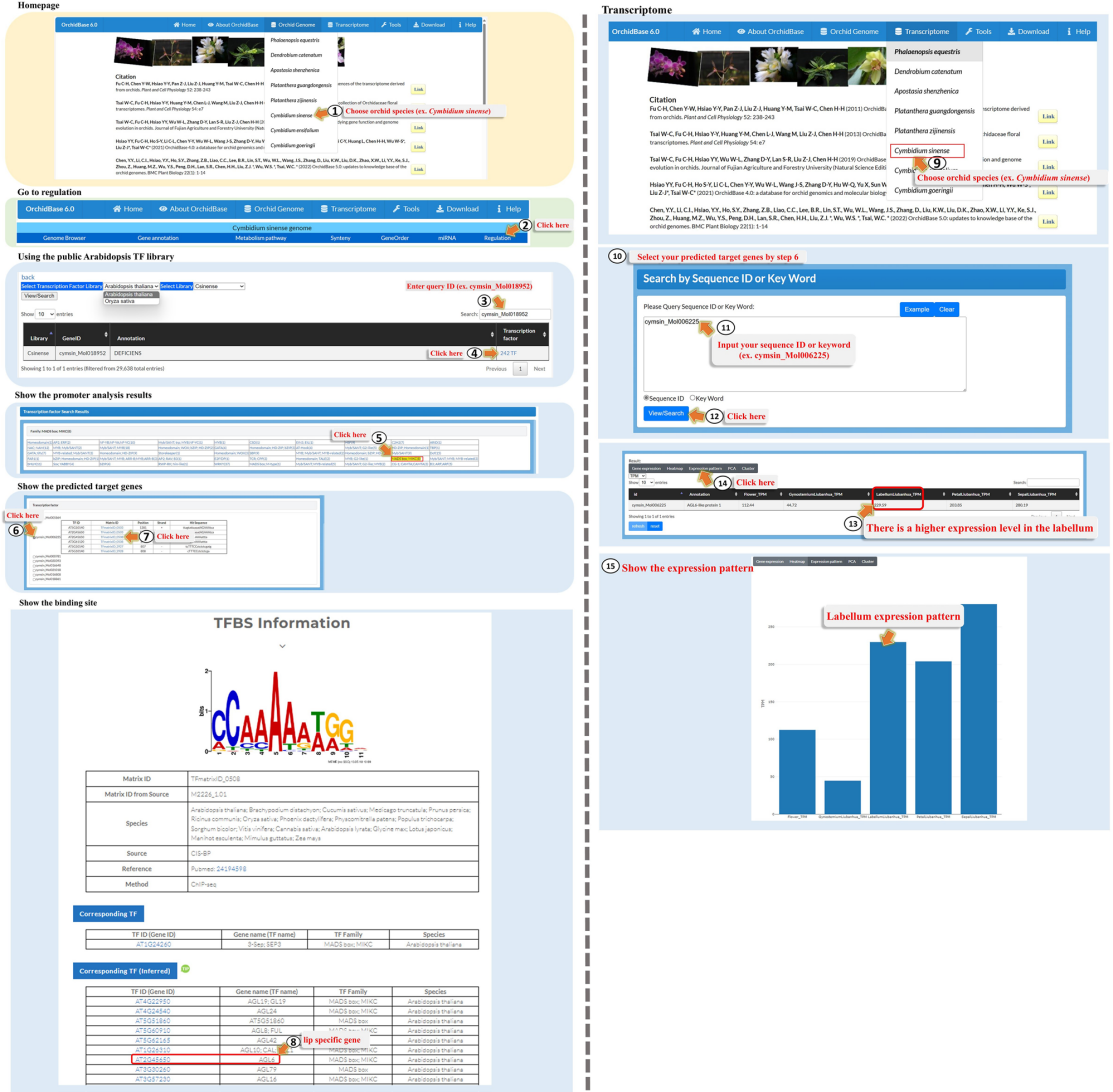
An example showing the use of the “Regulation” tool for analyzing transcription factors and their binding sites in the promoter of the *PeMADS4*-like gene in *C. sinense*

One of the most well-known orchid characteristics is their delicate floral organ labellum, which attracts pollinators for precision pollination and humans for art appreciation. Several models have been described for the MADS-box genes involved in labellum development, such as the Orchid Tepal Model [30], Orchid Code [31], the HOT (Homeotic Orchid Tepal) model [32], and P (perianth)-code [33]. One of the B-class MADS-box genes, *PeMADS4* in *Phalaenopsis*, has been proposed as a candidate labellum identity gene that is not excluded from the models. However, the genes that regulate the expression of *PeMADS4* orthologs in the labellum of orchids remain unclear. In this study, we demonstrated how to use ‘Regulation’ tool to screen TFs that could bind to the promoters of *PeMADS4* orthologs in *C. sinense*. First, we clicked “Orchid Genome”, chose one of the orchid species, *C. sinense* (Fig. 5, Step 1), and clicked “Regulation” (Fig. 5, Step 2). The ortholog ID (*cymsin_Mol018952*) of *PeMADS4* in *C. sinense* was identified using BLAST (data not shown). We then selected the “TF Reference Library” as *A. thaliana* and selected the library as *C. sinense*, or directly entered *cymsin_Mol018952* as the gene ID in the “Search” box (Fig. 5, Step 3). This page shows the annotation of *cymsin_Mol018952* and the number (242) of TFs that are possible regulators of *cymsin_Mol018952* expression. We clicked the 242 TFs (Fig. 5, Step 4), and then “MADS box, MIKC(8)” (Fig. 5, Step 5), because a previous study reported that MADS-box genes also have the ability to regulate the expression of self- or other MADS-box genes [32]. After choosing the second *Cymbidium* MADS-box gene *cymsin_Mol006225* (Fig. 5, Step 6), and clicking the third matrix ID “TFmatrixID_0508” (Fig. 5, Step 7), we could see the page linked to PlantPan 3.0 showing the binding logo and *Arabidopsis* TFs binding to it (see Fig. 8).

## Conclusions and future directions

We added the whole-genome sequences of the *Cymbidium* species, *C. sinense*, *C. ensifolium*, and *C. goeringii*, and their transcriptomes to OrchidBase 6.0. Additionally, two functions for genome comparisons and miRNA characterization were developed in this study. These additions have increased the number of *Cymbidium* genomes in OrchidBase and provided tools for exploring the knowledge embedded in nucleotide sequences. For instance, they offer accurate sequence data for CRISPR/Cas9 (Clustered Regularly Interspaced Short Palindromic Repeat/CRISPR-associated protein 9) technology for editing orchid genomes. Furthermore, the stored *Cymbidium* genomes present an opportunity for users to gain novel insights into the genome-wide effects on microevolution, aiding our understanding of the conservation and diversity of *Cymbidium* orchids. The genome sequences of new orchids are still being decoded, and novel biological analytical data is continually emerging. We will continue to focus on advanced orchid research and increase the variety of omics data in the OrchidBase.

## Electronic supplementary material

Below is the link to the electronic supplementary material.

Supplementary Material 1

## Data Availability

The raw data and whole genome-assembled scaffold sequences of the C. sinense and C. goeringii (PRJNA743748 and PRJNA749652) were downloaded from the National Center for Biotechnology Information (NCBI) database. The related genomic data of C. ensifolium (BioProject/GSA PRJCA005355/CRA004327) was retrieved from the National Genomics Data Center (NGDC). The transcriptomics data derived from the three Cymbidium species were also downloaded from BioProjects PRJNA743748, PRJNA749652, and BioProject/GSA PRJCA005355/CRA004327.
