# Supplementary material for: OrchidBase 6.0: increasing the number of Cymbidium (Orchidaceae) genomes and new bioinformatic tools for orchid genome analysis
Source: BMC Plant Biol. 2025 Jan 2;25:1. doi: 10.1186/s12870-024-06024-1 (PMC11697506; doi:10.1186/s12870-024-06024-1)
Supplement: Supplementary file 1 — Supplementary Material 1 [file 12870_2024_6024_MOESM1_ESM.docx]

Supplementary Table 1 Number of predicted transcription factors in each orchid genome, categorized by subfamily

| TF subfamily | *Asp. shenzhenica* | *P. zijinensis* | *P. guangdongensis* | *Pha. equestris* | *D. catenatum* | *C. sinense* | *C. ensifolium* | *C. goeringii* |
| --- | --- | --- | --- | --- | --- | --- | --- | --- |
| AP2 | 14 | 11 | 9 | 15 | 16 | 12 | 16 | 20 |
| ARF | 16 | 15 | 13 | 13 | 19 | 13 | 12 | 13 |
| ARR-B | 5 | 3 | 4 | 6 | 7 | 5 | 8 | 12 |
| B3 | 21 | 17 | 17 | 28 | 29 | 28 | 23 | 23 |
| BBR-BPC | 6 | 7 | 8 | 6 | 6 | 7 | 7 | 7 |
| BES1 | 6 | 6 | 6 | 6 | 7 | 7 | 8 | 9 |
| C2H2 | 81 | 72 | 62 | 92 | 116 | 130 | 143 | 98 |
| C3H | 37 | 35 | 43 | 39 | 46 | 58 | 53 | 72 |
| CAMTA | 4 | 4 | 6 | 4 | 7 | 3 | 6 | 8 |
| CO-like | 9 | 6 | 2 | 6 | 9 | 9 | 10 | 8 |
| CPP | 6 | 3 | 3 | 6 | 4 | 5 | 2 | 7 |
| DBB | 7 | 2 | 4 | 5 | 4 | 8 | 8 | 7 |
| Dof | 23 | 21 | 24 | 23 | 30 | 31 | 34 | 27 |
| E2F/DP | 6 | 7 | 8 | 5 | 6 | 12 | 13 | 11 |
| EIL | 4 | 6 | 7 | 4 | 4 | 3 | 4 | 2 |
| ERF | 93 | 64 | 59 | 85 | 93 | 102 | 113 | 84 |
| FAR1 | 24 | 138 | 8 | 26 | 23 | 18 | 24 | 24 |
| G2-like | 38 | 38 | 27 | 46 | 51 | 56 | 42 | 51 |
| GATA | 19 | 18 | 16 | 22 | 22 | 24 | 25 | 25 |
| GRAS | 51 | 47 | 39 | 49 | 49 | 57 | 57 | 55 |
| GRF | 7 | 10 | 9 | 9 | 10 | 10 | 11 | 11 |
| GeBP | 5 | 4 | 4 | 10 | 8 | 8 | 7 | 16 |
| HB-PHD | 2 | 1 | 1 | 0 | 1 | 1 | 2 | 1 |
| HB-other | 10 | 12 | 11 | 10 | 10 | 8 | 6 | 12 |
| HD-ZIP | 36 | 36 | 26 | 33 | 34 | 38 | 35 | 39 |
| HRT-like | 1 | 1 | 1 | 1 | 1 | 2 | 2 | 1 |
| HSF | 15 | 18 | 12 | 19 | 18 | 24 | 25 | 26 |
| LBD | 29 | 21 | 22 | 33 | 27 | 35 | 38 | 27 |
| LFY | 2 | 1 | 1 | 1 | 1 | 2 | 2 | 2 |
| LSD | 3 | 3 | 4 | 4 | 5 | 4 | 6 | 7 |
| M-type_MADS | 16 | 25 | 36 | 29 | 34 | 74 | 85 | 71 |
| MIKC_MADS | 21 | 19 | 15 | 22 | 29 | 25 | 31 | 22 |
| MYB | 74 | 76 | 69 | 104 | 105 | 106 | 116 | 100 |
| MYB_related | 54 | 44 | 51 | 63 | 58 | 57 | 58 | 77 |
| NAC | 62 | 57 | 52 | 83 | 85 | 94 | 95 | 89 |
| NF-X1 | 2 | 3 | 3 | 2 | 3 | 1 | 3 | 1 |
| NF-YA | 7 | 5 | 5 | 6 | 6 | 7 | 4 | 11 |
| NF-YB | 12 | 10 | 8 | 10 | 13 | 11 | 13 | 24 |
| NF-YC | 10 | 8 | 10 | 14 | 20 | 15 | 18 | 19 |
| NZZ/SPL | 1 | 0 | 0 | 0 | 0 | 0 | 0 | 1 |
| Nin-like | 5 | 3 | 5 | 6 | 4 | 8 | 8 | 4 |
| RAV | 4 | 3 | 3 | 4 | 2 | 3 | 3 | 2 |
| S1Fa-like | 2 | 0 | 1 | 1 | 1 | 1 | 2 | 2 |
| SAP | 0 | 3 | 2 | 0 | 0 | 1 | 1 | 1 |
| SBP | 18 | 11 | 11 | 15 | 15 | 18 | 19 | 18 |
| SRS | 5 | 3 | 2 | 4 | 5 | 7 | 5 | 6 |
| STAT | 1 | 1 | 1 | 1 | 1 | 1 | 0 | 0 |
| TALE | 15 | 15 | 16 | 16 | 19 | 21 | 25 | 22 |
| TCP | 21 | 12 | 14 | 22 | 25 | 15 | 14 | 15 |
| Trihelix | 27 | 23 | 22 | 31 | 33 | 29 | 33 | 28 |
| VOZ | 1 | 2 | 2 | 1 | 1 | 1 | 1 | 2 |
| WOX | 10 | 9 | 9 | 12 | 9 | 9 | 12 | 8 |
| WRKY | 42 | 47 | 43 | 62 | 68 | 64 | 72 | 69 |
| Whirly | 3 | 2 | 0 | 2 | 3 | 1 | 3 | 5 |
| YABBY | 7 | 6 | 4 | 10 | 10 | 8 | 7 | 12 |
| ZF-HD | 9 | 10 | 8 | 13 | 18 | 18 | 26 | 18 |
| bHLH | 88 | 89 | 74 | 97 | 102 | 114 | 112 | 134 |
| bZIP | 53 | 52 | 48 | 60 | 62 | 58 | 68 | 69 |
| Total Number | 1150 | 1165 | 970 | 1296 | 1394 | 1487 | 1576 | 1535 |
